# Crystal structure of (pyridine-κ*N*)bis(quinolin-2-olato-κ^2^ *N*,*O*)copper(II) monohydrate

**DOI:** 10.1107/S2056989015001279

**Published:** 2015-01-28

**Authors:** Benjamin Hawks, Jingjing Yan, Prem Basa, Shawn Burdette

**Affiliations:** a Department of Chemistry and Biochemistry, Worcester Polytechnic Institute, 100 Institute Road, Worcester, Massachusetts 01609-2280, USA

**Keywords:** crystal structure, copper(II), quinolin-8-ol, pyridine, hydrogen bonding

## Abstract

The title complex, [Cu(C_9_H_6_NO)_2_(C_5_H_4_N)]·H_2_O, adopts a slightly distorted square-pyramidal geometry in which the axial pyridine ligand exhibits a long Cu—N bond of 2.305 (3) Å. The pyridine ligand forms dihedral angles of 79.5 (5) and 88.0 (1)° with the planes of the two quinolin-2-olate ligands, while the dihedral angle between the quinoline groups of 9.0 (3)° indicates near planarity. The water mol­ecule connects adjacent copper complexes through O—H⋯O hydrogen bonds to phenolate O atoms, forming a network inter­connecting all the complexes in the crystal lattice.

## Related literature   

For the biological activity of clioquinol, see: Di Vaira *et al.* (2004 ▸). For the use of clioquinol in the treatment of Alzheimer’s disease, see: Bareggi & Cornelli (2012 ▸). For crystal structures of copper(II) complexes with 8-hy­droxy­quinoline (8-HQ) derivatives and the metal in a five-coordinate environment, see: Deraeve *et al.* (2008 ▸). For [Cu(8-HQ)_2_(H_2_O)_2_] with six-coordinate Cu(II), see: Okabe & Saishu (2001 ▸). For copper(II), zinc(II) and iron(III) crystalline complexes with 8-HQ, see: Palenik (1964 ▸); Najafi *et al.* (2011 ▸); Jian *et al.* (2001 ▸). For EPR studies performed on a putative [Cu(8-HQ)_2_(pyri­dine)] complex, see: Marov *et al.* (1975 ▸, 1978 ▸).
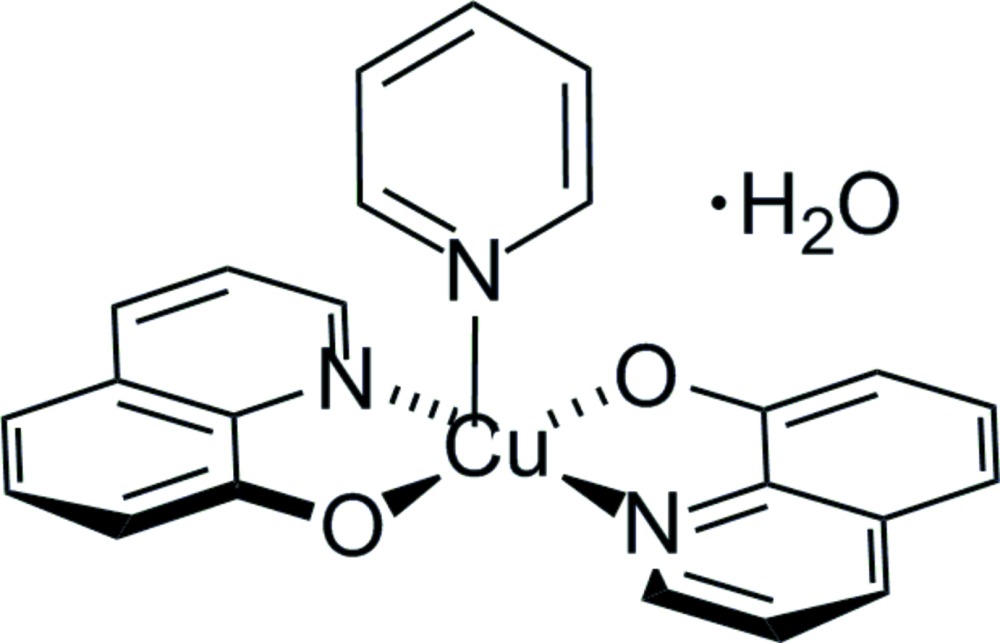


## Experimental   

### Crystal data  

[Cu(C_9_H_6_NO)_2_(C_5_H_5_N)]·H_2_O
*M* *_r_* = 448.95
Orthorhombic, 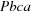

*a* = 8.9129 (4) Å
*b* = 13.9987 (7) Å
*c* = 32.2568 (16) Å
*V* = 4024.6 (3) Å^3^
*Z* = 8
Mo *K*α radiation
μ = 1.12 mm^−1^
*T* = 296 K
0.21 × 0.11 × 0.08 mm

### Data collection  

Bruker APEXII CCD diffractometer
Absorption correction: multi-scan (*SADABS*; Bruker, 2002 ▸) *T*_min_ = 0.800, *T*_max_ = 0.916
15390 measured reflections
3542 independent reflections
2301 reflections with *I* > 2σ(*I*)
*R*_int_ = 0.050

### Refinement  

*R*[*F* ^2^ > 2σ(*F* ^2^)] = 0.038
*wR*(*F* ^2^) = 0.093
*S* = 1.01
3542 reflections
279 parameters
H atoms treated by a mixture of independent and constrained refinement
Δρ_max_ = 0.24 e Å^−3^
Δρ_min_ = −0.27 e Å^−3^

### 

Data collection: *APEX2* (Bruker, 2002 ▸); cell refinement: *SAINT* (Bruker, 2002 ▸); data reduction: *SAINT*; program(s) used to solve structure: *SHELXS97* (Sheldrick, 2008 ▸); program(s) used to refine structure: *SHELXL97* (Sheldrick, 2015 ▸); molecular graphics: *SHELXTL* (Sheldrick, 2008 ▸); software used to prepare material for publication: *SHELXTL* and *publCIF* (Westrip, 2010 ▸).

## Supplementary Material

Crystal structure: contains datablock(s) I. DOI: 10.1107/S2056989015001279/jj2192sup1.cif

Structure factors: contains datablock(s) I. DOI: 10.1107/S2056989015001279/jj2192Isup2.hkl

Supporting information file. DOI: 10.1107/S2056989015001279/jj2192Isup3.cdx

**Fig. 1.**
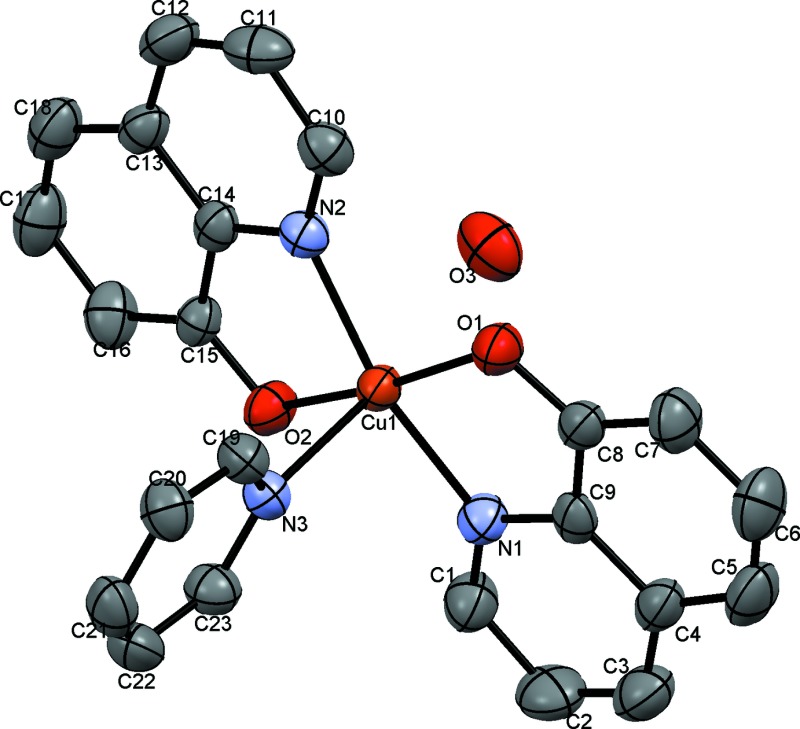
ORTEP drawing of [Cu(C9H6NO)2(C5H4N]·H2O. Displacement ellipsoids were drawn at the 50% probability level. The hydrogen atoms have been omitted for clarity.

9 6 2 5 4 2 . DOI: 10.1107/S2056989015001279/jj2192fig1.tif
ORTEP drawing of [Cu(C_9_H_6_NO)_2_(C_5_H_4_N]·H_2_O. Displacement ellipsoids were drawn at the 50% probability level. The hydrogen atoms have been omitted for clarity.

**Fig. 2.**
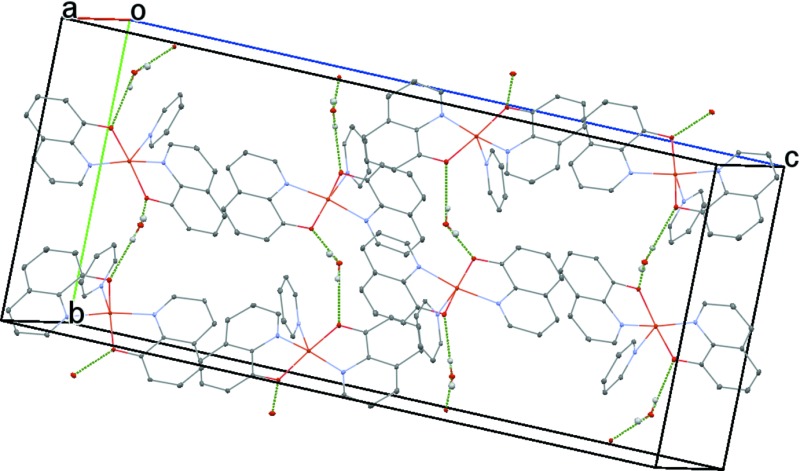
A portion of the molecular packing for [Cu(C9H6NO)2(C5H4N]·H2O viewed along the a axis. Dashed lines indicate O—H···O hydrogen bonds between water hydrogen atoms and hydroxyquinoline oxygen atoms forming continuous chains along the b axis. Displacement ellipsoids were drawn at the 5% probability level. Hydrogen atoms not involved in hydrogen bonds have been omitted for clarity.

9 6 2 5 4 2 a b . DOI: 10.1107/S2056989015001279/jj2192fig2.tif
A portion of the mol­ecular packing for [Cu(C_9_H_6_NO)_2_(C_5_H_4_N]·H_2_O viewed along the *a* axis. Dashed lines indicate O—H⋯O hydrogen bonds between water hydrogen atoms and hy­droxy­quinoline oxygen atoms forming continuous chains along the *b* axis. Displacement ellipsoids were drawn at the 5% probability level. Hydrogen atoms not involved in hydrogen bonds have been omitted for clarity.

CCDC reference: 1044628

Additional supporting information:  crystallographic information; 3D view; checkCIF report

## Figures and Tables

**Table:** 

Cu1O1	1.940(2)
Cu1O2	1.961(2)
Cu1N2	2.012(3)
Cu1N1	2.011(3)
Cu1N3	2.305(3)

**Table:** 

O1Cu1N2	92.81(10)
O2Cu1N2	83.32(9)
O1Cu1N1	84.23(10)
O2Cu1N1	97.30(10)
O1Cu1N3	97.70(9)
O2Cu1N3	91.41(9)
N2Cu1N3	100.91(10)
N1Cu1N3	94.18(10)

**Table 2:** Hydrogen-bond geometry (, )

*D*H*A*	*D*H	H*A*	*D* *A*	*D*H*A*
O3H18O2^i^	0.83(5)	1.95(5)	2.776(4)	173(4)
O3H19O1	0.76(4)	2.13(4)	2.871(4)	168(4)

Symmetry code: (i) 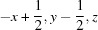.

## supplementary crystallographic information

### S1. Comment

Clioquinol is a 8-hy­droxy­quinoline (8-HQ) derivative that has been used for the treatment of Alzheimer's disease (Bareggi & Cornelli, 2012). The coordination chemistry of clioquinol plays a critical role in its biological activity (Di Vaira *et al.*, 2004). Copper(II), zinc(II) and iron(III) readily form crystalline complexes with 8-HQ and its derivatives (Palenik, 1964; Najafi, 2011; Jian *et al.*, 2001). Two bidentate ligands chelate a single metal ion in the square planar [Cu(8—HQ)_2_] cupric complex (Palenik, 1964) that is structurally analogous to [Cu(clioquinol)_2_] (Di Vaira *et al.*, 2004). Very few X-ray structures of five-coordinate cooper(II) complexes with 8-HQ derivatives have been reported (Deraeve *et al.*, 2008); however, a 6-coordinate complexe of [Cu(8—HQ)_2_(H_2_O)_2_] has been structurally characterized (Okabe & Saishu, 2001). EPR studies were performed on a putative [Cu(8—HQ)_2_(pyridine)] complex nearly 40 years ago (Marov *et al.*, 1975; Marov *et al.*, 1978).

The reaction of [Cu(OAc)_2_]·H_2_O with 8-hy­droxy­quinoline yields the well known [Cu(C_9_H_6_NO)_2_] moiety where each 8-hy­droxy­quionline group serves as a bidentate chelator coordinated through the oxygen and nitro­gen atoms. When recrystallized from a mixture of pyridine and H_2_O, the title complex is isolated. Herein, we report the crystal structure of [Cu(C_9_H_6_NO)_2_(C_5_H_4_N]·H_2_O, the first confirmation that the 5-coordinate complex can exist in the solid state. The pyridine ligand forms dihedral angles with the two quinolin-2-olate ligands of 79.5 (5)° and 88.0 (1)°, while the dihedral angle between the quinoline groups of 9.0 (3)° indicates near planarity. The Cu—N bond appears to be weak as the crystals readily decompose as they dry, presumably due to loss of the pyridine ligand.

### S2. Experimental

[Cu(OAc)_2_]·H_2_O (0.258 g, 1.29 mmol) and 8-hy­droxy­quinoline (0.375 g, 2.58 mol) were separately dissolved in minimal qu­anti­ties of acetic acid (ca. 2.5 mL). Upon mixing the solutions, a light green precipitate (0.486 g) formed immediately. The precipitate was isolated by filtration and dissolved in pyridine. Green blocks of [Cu(C_9_H_6_NO)_2_(C_5_H_4_N]·H_2_O were isolated by slow evaporation in a diffusion chamber containing water and subsequentally used for crystal structure analysis.

### S3. Refinement

H18 and H19 were located by a difference map and refined isotropically. All of the remaining H atoms were placed in calculated positions and refined using a riding model with atom–H lengths of 0.93 Å (CH). Isotropic displacement parameters for these atoms were set to 1.2 (CH) times U_eq_ of the parent atom.

### Crystal data

**Table:**

[Cu(C_9_H_6_NO)_2_(C_5_H_5_N)]·H_2_O	*F*(000) = 1848
*M**_r_* = 448.95	*D*_x_ = 1.482 Mg m^−^^3^
Orthorhombic, *P**b**c**a*	Mo *K*α radiation, λ = 0.71073 Å
Hall symbol: -P 2ac 2ab	Cell parameters from 2855 reflections
*a* = 8.9129 (4) Å	θ = 2.6–25.1°
*b* = 13.9987 (7) Å	µ = 1.12 mm^−^^1^
*c* = 32.2568 (16) Å	*T* = 296 K
*V* = 4024.6 (3) Å^3^	Block, green
*Z* = 8	0.21 × 0.11 × 0.08 mm

### Data collection

**Table:**

Bruker APEXII CCD diffractometer	3542 independent reflections
Radiation source: fine-focus sealed tube	2301 reflections with *I* > 2σ(*I*)
Graphite monochromator	*R*_int_ = 0.050
Detector resolution: 10.00 pixels mm^-1^	θ_max_ = 25.0°, θ_min_ = 2.6°
φ and ω scans	*h* = −10→9
Absorption correction: multi-scan (*SADABS*; Bruker, 2002)	*k* = −14→16
*T*_min_ = 0.800, *T*_max_ = 0.916	*l* = −27→38
15390 measured reflections	

### Refinement

**Table:**

Refinement on *F*^2^	Primary atom site location: structure-invariant direct methods
Least-squares matrix: full	Secondary atom site location: difference Fourier map
*R*[*F*^2^ > 2σ(*F*^2^)] = 0.038	Hydrogen site location: inferred from neighbouring sites
*wR*(*F*^2^) = 0.093	H atoms treated by a mixture of independent and constrained refinement
*S* = 1.01	*w* = 1/[σ^2^(*F*_o_^2^) + (0.0441*P*)^2^ + 0.2191*P*] where *P* = (*F*_o_^2^ + 2*F*_c_^2^)/3
3542 reflections	(Δ/σ)_max_ = 0.001
279 parameters	Δρ_max_ = 0.24 e Å^−^^3^
0 restraints	Δρ_min_ = −0.27 e Å^−^^3^

### Special details

**Table:**

Geometry. All e.s.d.'s (except the e.s.d. in the dihedral angle between two l.s. planes) are estimated using the full covariance matrix. The cell e.s.d.'s are taken into account individually in the estimation of e.s.d.'s in distances, angles and torsion angles; correlations between e.s.d.'s in cell parameters are only used when they are defined by crystal symmetry. An approximate (isotropic) treatment of cell e.s.d.'s is used for estimating e.s.d.'s involving l.s. planes.
Refinement. Refinement of *F*^2^ against ALL reflections. The weighted *R*-factor *wR* and goodness of fit *S* are based on *F*^2^, conventional *R*-factors *R* are based on *F*, with *F* set to zero for negative *F*^2^. The threshold expression of *F*^2^ > σ(*F*^2^) is used only for calculating *R*-factors(gt) *etc*. and is not relevant to the choice of reflections for refinement. *R*-factors based on *F*^2^ are statistically about twice as large as those based on *F*, and *R*- factors based on ALL data will be even larger.

### Fractional atomic coordinates and isotropic or equivalent isotropic displacement parameters (Å^2^)

**Table:**

	*x*	*y*	*z*	*U*_iso_*/*U*_eq_	
Cu1	0.16872 (4)	0.91228 (3)	0.376062 (11)	0.03289 (14)	
O1	0.0393 (2)	0.81502 (16)	0.39962 (6)	0.0436 (6)	
O2	0.2691 (2)	1.02164 (15)	0.35011 (6)	0.0415 (6)	
N1	0.1945 (3)	0.9539 (2)	0.43529 (8)	0.0393 (7)	
N2	0.0918 (3)	0.88951 (18)	0.31826 (8)	0.0346 (6)	
N3	0.3873 (3)	0.82435 (19)	0.37496 (8)	0.0362 (6)	
C1	0.2740 (4)	1.0222 (3)	0.45151 (10)	0.0498 (9)	
H1	0.3242	1.0640	0.4339	0.060*	
C2	0.2872 (4)	1.0353 (3)	0.49500 (11)	0.0630 (11)	
H6	0.3453	1.0847	0.5057	0.076*	
C3	0.2132 (4)	0.9742 (3)	0.52092 (11)	0.0573 (10)	
H2	0.2213	0.9815	0.5495	0.069*	
C4	0.1255 (4)	0.9008 (3)	0.50465 (10)	0.0438 (9)	
C5	0.0420 (4)	0.8341 (3)	0.52762 (11)	0.0592 (11)	
H5	0.0434	0.8369	0.5564	0.071*	
C6	−0.0403 (5)	0.7661 (3)	0.50858 (12)	0.0646 (12)	
H4	−0.0964	0.7241	0.5246	0.078*	
C7	−0.0436 (4)	0.7570 (3)	0.46570 (11)	0.0524 (10)	
H3	−0.1001	0.7086	0.4537	0.063*	
C8	0.0358 (4)	0.8188 (2)	0.44086 (9)	0.0383 (8)	
C9	0.1197 (4)	0.8923 (2)	0.46062 (10)	0.0360 (8)	
C10	0.0008 (4)	0.8235 (2)	0.30402 (10)	0.0429 (9)	
H7	−0.0425	0.7807	0.3225	0.051*	
C11	−0.0332 (4)	0.8157 (3)	0.26144 (11)	0.0534 (10)	
H12	−0.0974	0.7680	0.2521	0.064*	
C12	0.0284 (4)	0.8783 (3)	0.23423 (11)	0.0505 (10)	
H11	0.0063	0.8733	0.2061	0.061*	
C13	0.1255 (4)	0.9507 (2)	0.24823 (9)	0.0385 (8)	
C14	0.1537 (3)	0.9534 (2)	0.29164 (9)	0.0340 (8)	
C15	0.2494 (4)	1.0239 (2)	0.30931 (9)	0.0335 (8)	
C16	0.3130 (4)	1.0896 (2)	0.28342 (11)	0.0495 (9)	
H10	0.3749	1.1369	0.2943	0.059*	
C17	0.2858 (5)	1.0865 (3)	0.24070 (11)	0.0569 (11)	
H9	0.3312	1.1317	0.2237	0.068*	
C18	0.1952 (4)	1.0196 (3)	0.22332 (10)	0.0542 (10)	
H8	0.1794	1.0196	0.1948	0.065*	
C19	0.3876 (4)	0.7353 (2)	0.36165 (10)	0.0418 (9)	
H17	0.2954	0.7070	0.3560	0.050*	
C20	0.5146 (4)	0.6814 (3)	0.35556 (10)	0.0475 (9)	
H16	0.5084	0.6191	0.3457	0.057*	
C21	0.6507 (4)	0.7222 (3)	0.36438 (10)	0.0512 (10)	
H15	0.7393	0.6881	0.3607	0.061*	
C22	0.6536 (4)	0.8152 (3)	0.37883 (10)	0.0515 (9)	
H14	0.7441	0.8448	0.3852	0.062*	
C23	0.5208 (4)	0.8629 (3)	0.38358 (10)	0.0433 (9)	
H13	0.5236	0.9254	0.3933	0.052*	
O3	−0.0217 (4)	0.6211 (2)	0.37607 (10)	0.0597 (8)	
H19	0.006 (4)	0.671 (3)	0.3806 (12)	0.058 (15)*	
H18	0.054 (5)	0.595 (3)	0.3664 (13)	0.083 (17)*	

### Atomic displacement parameters (Å^2^)

**Table:**

	*U*^11^	*U*^22^	*U*^33^	*U*^12^	*U*^13^	*U*^23^
Cu1	0.0354 (2)	0.0357 (2)	0.0275 (2)	−0.00361 (19)	0.00111 (18)	−0.00006 (17)
O1	0.0458 (14)	0.0499 (14)	0.0351 (12)	−0.0102 (12)	0.0018 (11)	0.0002 (11)
O2	0.0508 (14)	0.0390 (13)	0.0348 (12)	−0.0096 (12)	0.0002 (11)	0.0031 (10)
N1	0.0405 (17)	0.0416 (17)	0.0357 (14)	−0.0023 (15)	0.0023 (13)	−0.0040 (13)
N2	0.0335 (16)	0.0333 (16)	0.0369 (15)	0.0002 (14)	−0.0009 (13)	−0.0004 (12)
N3	0.0314 (16)	0.0397 (16)	0.0374 (14)	0.0014 (13)	0.0003 (13)	−0.0009 (13)
C1	0.055 (2)	0.051 (2)	0.044 (2)	−0.006 (2)	0.0066 (19)	−0.0045 (18)
C2	0.063 (3)	0.069 (3)	0.058 (2)	−0.008 (2)	−0.010 (2)	−0.022 (2)
C3	0.064 (3)	0.069 (3)	0.039 (2)	0.006 (2)	−0.005 (2)	−0.009 (2)
C4	0.045 (2)	0.055 (2)	0.0315 (17)	0.0112 (19)	0.0017 (16)	−0.0007 (17)
C5	0.068 (3)	0.077 (3)	0.0323 (19)	0.012 (2)	0.006 (2)	0.012 (2)
C6	0.075 (3)	0.069 (3)	0.049 (2)	−0.007 (2)	0.016 (2)	0.015 (2)
C7	0.053 (3)	0.054 (3)	0.050 (2)	−0.0042 (19)	0.009 (2)	0.0111 (18)
C8	0.035 (2)	0.046 (2)	0.0330 (17)	0.0102 (18)	0.0003 (15)	0.0004 (16)
C9	0.0291 (19)	0.043 (2)	0.0363 (17)	0.0119 (16)	0.0047 (15)	0.0055 (15)
C10	0.039 (2)	0.041 (2)	0.049 (2)	−0.0005 (19)	−0.0002 (17)	0.0042 (17)
C11	0.050 (2)	0.050 (2)	0.060 (2)	−0.001 (2)	−0.018 (2)	−0.012 (2)
C12	0.056 (3)	0.061 (3)	0.0341 (18)	0.009 (2)	−0.0086 (19)	−0.0006 (18)
C13	0.041 (2)	0.0435 (19)	0.0304 (16)	0.0115 (18)	−0.0014 (16)	0.0001 (16)
C14	0.034 (2)	0.0360 (18)	0.0324 (16)	0.0095 (17)	0.0033 (15)	−0.0001 (15)
C15	0.037 (2)	0.0334 (18)	0.0302 (16)	0.0038 (16)	0.0040 (16)	−0.0008 (15)
C16	0.057 (2)	0.039 (2)	0.053 (2)	−0.0053 (19)	0.0119 (19)	0.0004 (17)
C17	0.071 (3)	0.054 (2)	0.046 (2)	0.003 (2)	0.015 (2)	0.0144 (19)
C18	0.063 (3)	0.063 (3)	0.0360 (19)	0.012 (2)	0.0049 (19)	0.0081 (19)
C19	0.033 (2)	0.050 (2)	0.0422 (19)	−0.0021 (19)	−0.0001 (15)	−0.0027 (17)
C20	0.045 (2)	0.044 (2)	0.053 (2)	0.005 (2)	0.0072 (19)	−0.0028 (17)
C21	0.039 (2)	0.063 (3)	0.051 (2)	0.012 (2)	0.0079 (18)	0.0066 (19)
C22	0.034 (2)	0.073 (3)	0.048 (2)	−0.003 (2)	−0.0066 (18)	0.005 (2)
C23	0.042 (2)	0.043 (2)	0.045 (2)	−0.0025 (19)	−0.0031 (17)	−0.0039 (16)
O3	0.0487 (19)	0.0478 (19)	0.083 (2)	0.0065 (16)	0.0051 (17)	−0.0096 (17)

### Geometric parameters (Å, º)

**Table:**

Cu1—O1	1.940 (2)	C8—C9	1.422 (4)
Cu1—O2	1.961 (2)	C10—C11	1.411 (4)
Cu1—N2	2.012 (3)	C10—H7	0.9300
Cu1—N1	2.011 (3)	C11—C12	1.356 (5)
Cu1—N3	2.305 (3)	C11—H12	0.9300
O1—C8	1.332 (3)	C12—C13	1.408 (5)
O2—C15	1.328 (3)	C12—H11	0.9300
N1—C1	1.299 (4)	C13—C18	1.401 (5)
N1—C9	1.363 (4)	C13—C14	1.423 (4)
N2—C10	1.312 (4)	C14—C15	1.424 (4)
N2—C14	1.357 (4)	C15—C16	1.366 (4)
N3—C19	1.318 (4)	C16—C17	1.400 (5)
N3—C23	1.336 (4)	C16—H10	0.9300
C1—C2	1.420 (4)	C17—C18	1.357 (5)
C1—H1	0.9300	C17—H9	0.9300
C2—C3	1.366 (5)	C18—H8	0.9300
C2—H6	0.9300	C19—C20	1.375 (4)
C3—C4	1.393 (5)	C19—H17	0.9300
C3—H2	0.9300	C20—C21	1.371 (4)
C4—C5	1.405 (5)	C20—H16	0.9300
C4—C9	1.426 (4)	C21—C22	1.383 (5)
C5—C6	1.349 (5)	C21—H15	0.9300
C5—H5	0.9300	C22—C23	1.367 (4)
C6—C7	1.389 (5)	C22—H14	0.9300
C6—H4	0.9300	C23—H13	0.9300
C7—C8	1.375 (4)	O3—H19	0.76 (4)
C7—H3	0.9300	O3—H18	0.83 (5)
			
O1—Cu1—O2	170.64 (9)	N1—C9—C4	121.8 (3)
O1—Cu1—N2	92.81 (10)	C8—C9—C4	121.7 (3)
O2—Cu1—N2	83.32 (9)	N2—C10—C11	121.8 (3)
O1—Cu1—N1	84.23 (10)	N2—C10—H7	119.1
O2—Cu1—N1	97.30 (10)	C11—C10—H7	119.1
N2—Cu1—N1	164.89 (11)	C12—C11—C10	119.6 (3)
O1—Cu1—N3	97.70 (9)	C12—C11—H12	120.2
O2—Cu1—N3	91.41 (9)	C10—C11—H12	120.2
N2—Cu1—N3	100.91 (10)	C11—C12—C13	120.4 (3)
N1—Cu1—N3	94.18 (10)	C11—C12—H11	119.8
C8—O1—Cu1	112.2 (2)	C13—C12—H11	119.8
C15—O2—Cu1	112.42 (19)	C18—C13—C12	125.8 (3)
C1—N1—C9	119.4 (3)	C18—C13—C14	117.9 (3)
C1—N1—Cu1	131.2 (2)	C12—C13—C14	116.3 (3)
C9—N1—Cu1	109.3 (2)	N2—C14—C15	116.6 (3)
C10—N2—C14	119.6 (3)	N2—C14—C13	122.2 (3)
C10—N2—Cu1	130.3 (2)	C15—C14—C13	121.2 (3)
C14—N2—Cu1	110.1 (2)	O2—C15—C16	124.6 (3)
C19—N3—C23	116.6 (3)	O2—C15—C14	117.3 (3)
C19—N3—Cu1	120.8 (2)	C16—C15—C14	118.1 (3)
C23—N3—Cu1	122.3 (2)	C15—C16—C17	120.6 (3)
N1—C1—C2	122.6 (4)	C15—C16—H10	119.7
N1—C1—H1	118.7	C17—C16—H10	119.7
C2—C1—H1	118.7	C18—C17—C16	122.1 (3)
C3—C2—C1	118.9 (4)	C18—C17—H9	119.0
C3—C2—H6	120.6	C16—C17—H9	119.0
C1—C2—H6	120.6	C17—C18—C13	120.1 (3)
C2—C3—C4	120.1 (3)	C17—C18—H8	119.9
C2—C3—H2	119.9	C13—C18—H8	119.9
C4—C3—H2	119.9	N3—C19—C20	124.5 (3)
C3—C4—C5	126.0 (3)	N3—C19—H17	117.7
C3—C4—C9	117.2 (3)	C20—C19—H17	117.7
C5—C4—C9	116.8 (3)	C21—C20—C19	118.1 (3)
C6—C5—C4	121.1 (3)	C21—C20—H16	121.0
C6—C5—H5	119.5	C19—C20—H16	121.0
C4—C5—H5	119.5	C20—C21—C22	118.6 (3)
C5—C6—C7	122.0 (4)	C20—C21—H15	120.7
C5—C6—H4	119.0	C22—C21—H15	120.7
C7—C6—H4	119.0	C23—C22—C21	118.8 (3)
C8—C7—C6	120.8 (4)	C23—C22—H14	120.6
C8—C7—H3	119.6	C21—C22—H14	120.6
C6—C7—H3	119.6	N3—C23—C22	123.4 (3)
O1—C8—C7	124.7 (3)	N3—C23—H13	118.3
O1—C8—C9	117.7 (3)	C22—C23—H13	118.3
C7—C8—C9	117.7 (3)	H19—O3—H18	102 (4)
N1—C9—C8	116.5 (3)		
			
N2—Cu1—O1—C8	−168.2 (2)	Cu1—N1—C9—C8	−3.6 (3)
N1—Cu1—O1—C8	−3.1 (2)	C1—N1—C9—C4	0.3 (5)
N3—Cu1—O1—C8	90.4 (2)	Cu1—N1—C9—C4	176.2 (2)
N2—Cu1—O2—C15	−4.6 (2)	O1—C8—C9—N1	1.2 (4)
N1—Cu1—O2—C15	−169.4 (2)	C7—C8—C9—N1	−178.5 (3)
N3—Cu1—O2—C15	96.2 (2)	O1—C8—C9—C4	−178.6 (3)
O1—Cu1—N1—C1	178.9 (3)	C7—C8—C9—C4	1.7 (5)
O2—Cu1—N1—C1	−10.4 (3)	C3—C4—C9—N1	−1.0 (5)
N2—Cu1—N1—C1	−101.8 (5)	C5—C4—C9—N1	179.0 (3)
N3—Cu1—N1—C1	81.6 (3)	C3—C4—C9—C8	178.8 (3)
O1—Cu1—N1—C9	3.6 (2)	C5—C4—C9—C8	−1.2 (5)
O2—Cu1—N1—C9	174.3 (2)	C14—N2—C10—C11	1.1 (5)
N2—Cu1—N1—C9	82.9 (5)	Cu1—N2—C10—C11	−176.0 (2)
N3—Cu1—N1—C9	−93.7 (2)	N2—C10—C11—C12	−0.5 (5)
O1—Cu1—N2—C10	−7.0 (3)	C10—C11—C12—C13	−0.1 (5)
O2—Cu1—N2—C10	−178.4 (3)	C11—C12—C13—C18	−179.6 (3)
N1—Cu1—N2—C10	−85.2 (5)	C11—C12—C13—C14	0.1 (5)
N3—Cu1—N2—C10	91.4 (3)	C10—N2—C14—C15	179.1 (3)
O1—Cu1—N2—C14	175.7 (2)	Cu1—N2—C14—C15	−3.3 (3)
O2—Cu1—N2—C14	4.3 (2)	C10—N2—C14—C13	−1.1 (4)
N1—Cu1—N2—C14	97.5 (5)	Cu1—N2—C14—C13	176.5 (2)
N3—Cu1—N2—C14	−85.9 (2)	C18—C13—C14—N2	−179.8 (3)
O1—Cu1—N3—C19	44.9 (2)	C12—C13—C14—N2	0.5 (5)
O2—Cu1—N3—C19	−133.0 (2)	C18—C13—C14—C15	0.1 (5)
N2—Cu1—N3—C19	−49.5 (3)	C12—C13—C14—C15	−179.6 (3)
N1—Cu1—N3—C19	129.6 (2)	Cu1—O2—C15—C16	−176.7 (3)
O1—Cu1—N3—C23	−142.0 (2)	Cu1—O2—C15—C14	4.2 (3)
O2—Cu1—N3—C23	40.1 (2)	N2—C14—C15—O2	−0.5 (4)
N2—Cu1—N3—C23	123.6 (2)	C13—C14—C15—O2	179.7 (3)
N1—Cu1—N3—C23	−57.3 (2)	N2—C14—C15—C16	−179.7 (3)
C9—N1—C1—C2	0.3 (5)	C13—C14—C15—C16	0.5 (5)
Cu1—N1—C1—C2	−174.5 (3)	O2—C15—C16—C17	−179.9 (3)
N1—C1—C2—C3	−0.3 (6)	C14—C15—C16—C17	−0.8 (5)
C1—C2—C3—C4	−0.5 (6)	C15—C16—C17—C18	0.6 (6)
C2—C3—C4—C5	−178.9 (4)	C16—C17—C18—C13	0.0 (6)
C2—C3—C4—C9	1.1 (5)	C12—C13—C18—C17	179.4 (3)
C3—C4—C5—C6	179.5 (4)	C14—C13—C18—C17	−0.3 (5)
C9—C4—C5—C6	−0.5 (5)	C23—N3—C19—C20	−1.1 (5)
C4—C5—C6—C7	1.7 (6)	Cu1—N3—C19—C20	172.4 (2)
C5—C6—C7—C8	−1.1 (6)	N3—C19—C20—C21	0.9 (5)
Cu1—O1—C8—C7	−178.3 (3)	C19—C20—C21—C22	−0.1 (5)
Cu1—O1—C8—C9	2.0 (3)	C20—C21—C22—C23	−0.3 (5)
C6—C7—C8—O1	179.8 (3)	C19—N3—C23—C22	0.7 (5)
C6—C7—C8—C9	−0.5 (5)	Cu1—N3—C23—C22	−172.7 (2)
C1—N1—C9—C8	−179.5 (3)	C21—C22—C23—N3	0.0 (5)

### Hydrogen-bond geometry (Å, º)

**Table:**

*D*—H···*A*	*D*—H	H···*A*	*D*···*A*	*D*—H···*A*
O3—H18···O2^i^	0.83 (5)	1.95 (5)	2.776 (4)	173 (4)
O3—H19···O1	0.76 (4)	2.13 (4)	2.871 (4)	168 (4)

Symmetry code: (i) −*x*+1/2, *y*−1/2, *z*.
