# Supplementary figures and images for: Crystal structure of (pyridine-κN)bis(quinolin-2-olato-κ2 N,O)copper(II) monohydrate
Source: Acta Crystallogr E Crystallogr Commun. 2015 Jan 28;71(Pt 2):m38–9. doi: 10.1107/S2056989015001279 (PMC4384625; doi:10.1107/S2056989015001279)

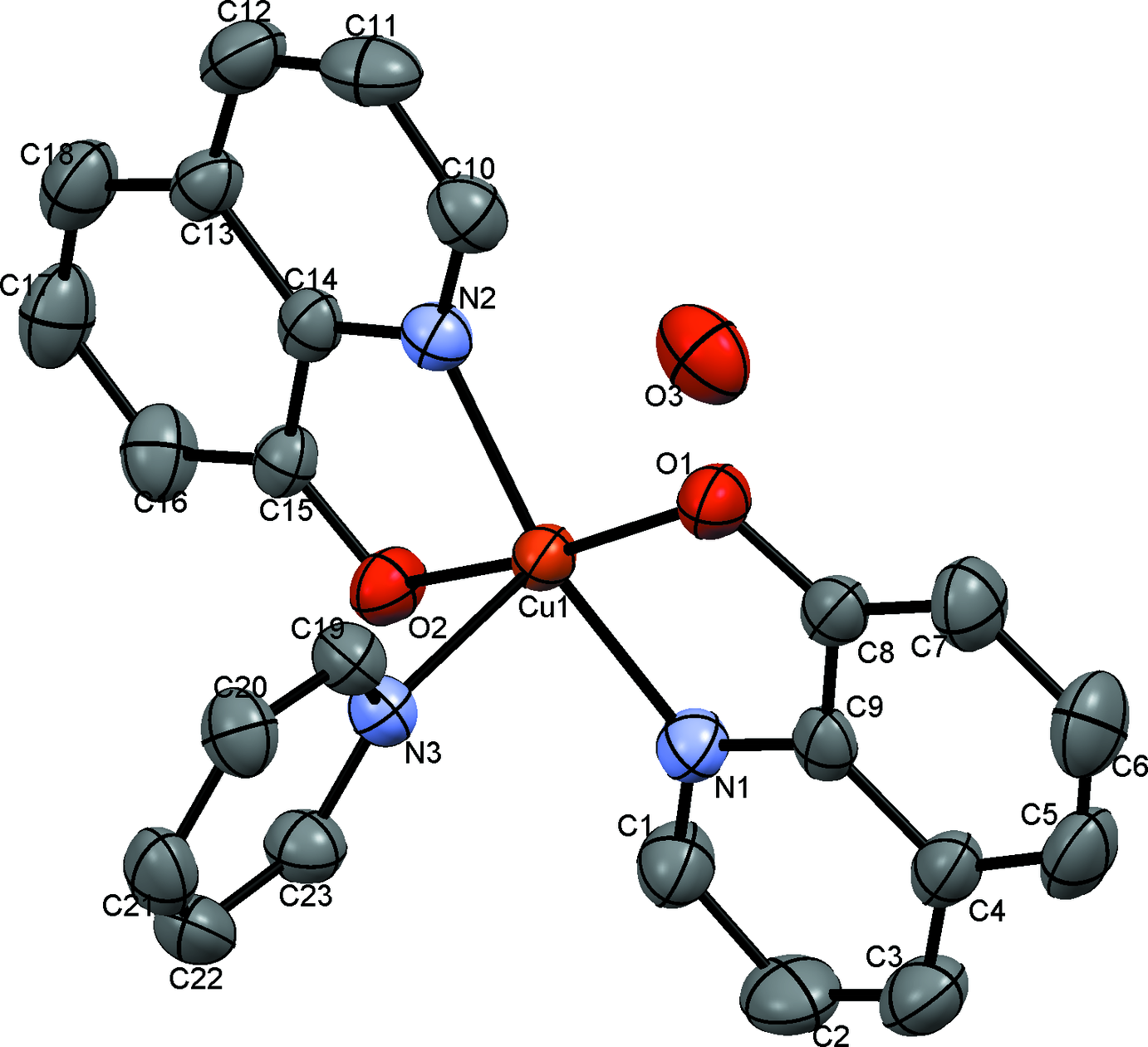

Supplement: Supplementary file 4 [file e-71-00m38-fig1.tif]

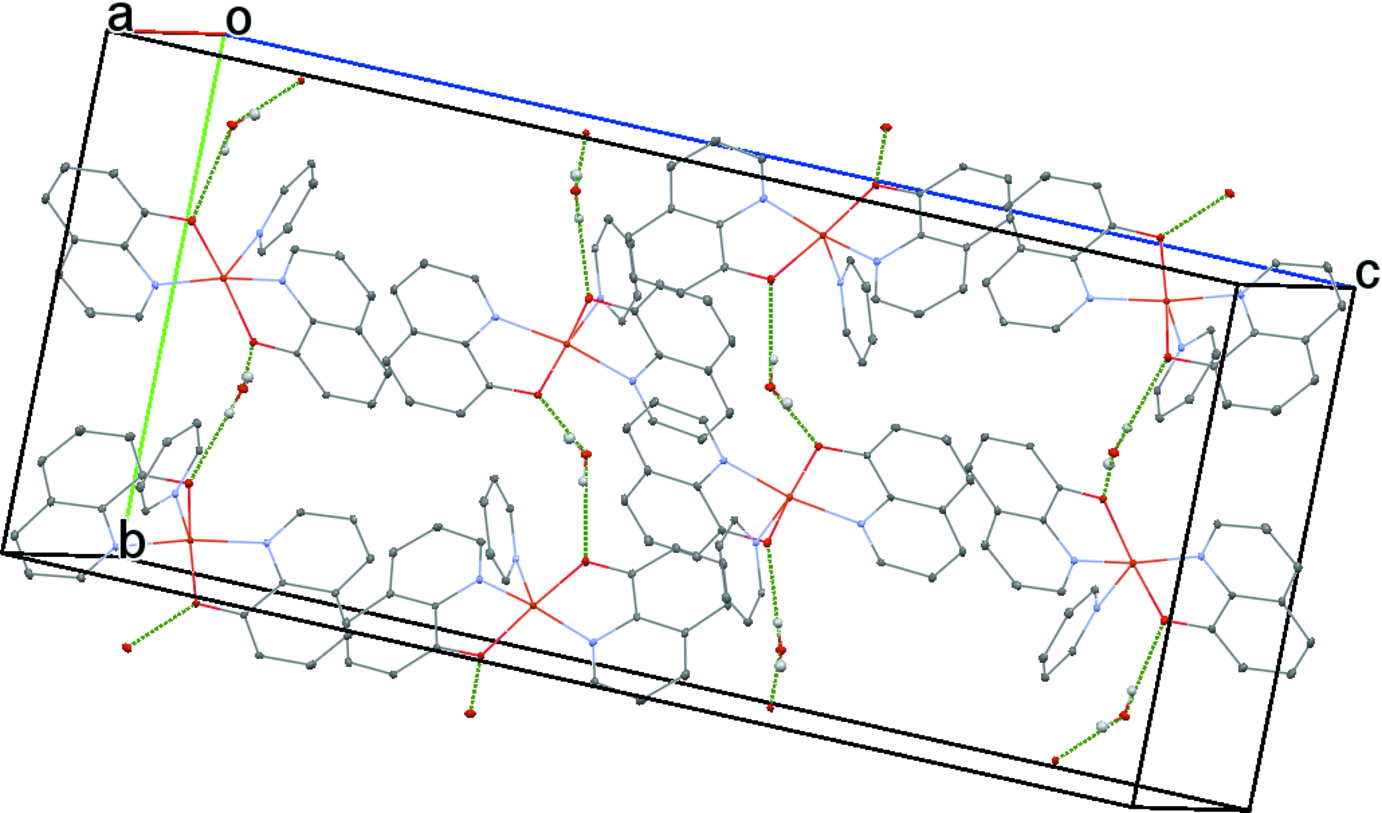

Supplement: Supplementary file 5 [file e-71-00m38-fig2.tif]
